# Venetoclax is a potent hepsin inhibitor that reduces the metastatic and prothrombotic phenotypes of hepsin-expressing colorectal cancer cells

**DOI:** 10.3389/fmolb.2023.1182925

**Published:** 2023-05-19

**Authors:** Maria Carmen Rodenas, Julia Peñas-Martínez, Irene Pardo-Sánchez, David Zaragoza-Huesca, Carmen Ortega-Sabater, Jorge Peña-García, Salvador Espín, Guillermo Ricote, Sofía Montenegro, Francisco Ayala-De La Peña, Ginés Luengo-Gil, Andrés Nieto, Francisco García-Molina, Vicente Vicente, Francesco Bernardi, María Luisa Lozano, Victoriano Mulero, Horacio Pérez-Sánchez, Alberto Carmona-Bayonas, Irene Martínez-Martínez

**Affiliations:** ^1^ Department of Hematology and Medical Oncology, Hospital Universitario Morales Meseguer, Centro Regional de Hemodonación, Centro de Investigación Biomédica en Red de Enfermedades Raras, IMIB-Pascual Parrilla, Universidad de Murcia, Murcia, Spain; ^2^ Department of Cell Biology, Faculty of Biology, Centro de Investigación Biomédica en Red de Enfermedades Raras, IMIB-Pascual Parrilla, Universidad de Murcia, Murcia, Spain; ^3^ Computer Engineering Department, Structural Bioinformatics and High Performance Computing Research Group (BIO-HPC), UCAM Universidad Católica de Murcia, Guadalupe, Spain; ^4^ Clinical Analysis and Pathology Department, Group of Molecular Pathology and Pharmacogenetics, IMIB-Pascual Parrilla, Hospital Universitario Santa Lucía, Cartagena, Spain; ^5^ Department of Pathology, Hospital Universitario Morales Meseguer, Murcia, Spain; ^6^ Department of Pathology, Hospital Universitario Reina Sofía, Murcia, Spain; ^7^ Department of Life Sciences and Biotechnology, University of Ferrara, Ferrara, Italy

**Keywords:** hepsin, colorectal cancer, metastasis, thrombosis, molecular-targeted therapy

## Abstract

**Introduction:** Hepsin is a type II transmembrane serine protease and its expression has been linked to greater tumorigenicity and worse prognosis in different tumors. Recently, our group demonstrated that high hepsin levels from primary tumor were associated with a higher risk of metastasis and thrombosis in localized colorectal cancer patients. This study aims to explore the molecular role of hepsin in colorectal cancer.

**Methods:** Hepsin levels in plasma from resected and metastatic colorectal cancer patients were analyzed by ELISA. The effect of hepsin levels on cell migration, invasion, and proliferation, as well as on the activation of crucial cancer signaling pathways, was performed *in vitro* using colorectal cancer cells. A thrombin generation assay determined the procoagulant function of hepsin from these cells. A virtual screening of a database containing more than 2000 FDA-approved compounds was performed to screen hepsin inhibitors, and selected compounds were tested *in vitro* for their ability to suppress hepsin effects in colorectal cancer cells. Xenotransplantation assays were done in zebrafish larvae to study the impact of venetoclax on invasion promoted by hepsin.

**Results:** Our results showed higher plasma hepsin levels in metastatic patients, among which, hepsin was higher in those suffering thrombosis. Hepsin overexpression increased colorectal cancer cell invasion, Erk1/2 and STAT3 phosphorylation, and thrombin generation in plasma. In addition, we identified venetoclax as a potent hepsin inhibitor that reduced the metastatic and prothrombotic phenotypes of hepsin-expressing colorectal cancer cells. Interestingly, pretreatment with Venetoclax of cells overexpressing hepsin reduced their invasiveness *in vivo*.

**Discussion:** Our results demonstrate that hepsin overexpression correlates with a more aggressive and prothrombotic tumor phenotype. Likewise, they demonstrate the antitumor role of venetoclax as a hepsin inhibitor, laying the groundwork for molecular-targeted therapy for colorectal cancer.

## 1 Introduction

Proteolysis in the tumor microenvironment confers an adaptive advantage to emerging tumors through its capacity to remodel the extracellular matrix and orchestrate various processes, such as angiogenesis, invasion and metastasis. Multiple proteases are deregulated in tumor cells to promote their progression and spread (Damalanka and Janetka, 2019). However, not all tumors develop the same strategies nor do they activate the expression of the same proteases to sustain tumorigenic processes. Type II transmembrane serine proteases (TTSP) comprise a family whose deregulation is involved in these processes (Damalanka and Janetka, 2019). Hepsin is a TTSP whose expression has been linked to greater tumorigenicity in different types of cancer, such as breast, lung, prostate and gastric cancer (Zhang et al., 2016; Damalanka et al., 2021; Tervonen et al., 2021; Lu et al., 2022). It has been described that hepsin activates extracellular ligands that promote cancer progression, such as hepatocyte-growth factor (Li et al., 2020; Fu et al., 2021). In addition, this TTSP degrades and remodels extracellular matrix, disrupting epithelial integrity and thus increasing tumor invasion (Tanabe and List, 2017). In this context, hepsin is a key protease in Ras-dependent tumorigenesis that affects epithelial cohesion and basement membrane integrity (Tervonen et al., 2021). This could be relevant in tumors with a high prevalence of Ras mutations, such as colorectal cancer (CRC) (Bylsma et al., 2020), where the implication of hepsin is practically unknown. Recently, our group demonstrated that a high hepsin expression in the primary tumor increased metastasis risk in localized CRC patients (Zaragoza-Huesca et al., 2022). In addition, hepsin is a serum marker that can distinguish between localized and metastatic CRC (Tieng et al., 2020). However, functional mechanisms underlying these findings in CRC have not been studied yet.

Curiously, hepsin effects in cancer are not only tumorigenic, but its role is multifaceted (Halabian et al., 2009; Khandekar and Jagadeeswaran, 2014; Bylsma et al., 2020; Tieng et al., 2020). Thus, some articles describe high hepsin levels associated with well-differentiated tumors, antitumor activity, and a better prognosis. This phenomenon is known as “the hepsin paradox” (Halabian et al., 2009; Kim et al., 2020; Mahajan et al., 2022). Our group found similar results in the biopsies of patients with CRC. Thus, on the one hand, hepsin expression reduced disease-free survival in localized patients.

In contrast, on the other hand, in metastatic patients, low hepsin expression was observed in poorly differentiated tumors and in patients where a larger number of distant organs were affected (Zaragoza-Huesca et al., 2022). In addition to its multifaceted tumor effect, hepsin has another peculiarity—it activates coagulation factor VII, which initiates the extrinsic coagulation cascade. Hepsin’s involvement in activation of the coagulation has been proven *in vitro* and zebrafish models (Halabian et al., 2009; Khandekar and Jagadeeswaran, 2014). Recently, we showed that hepsin from primary tumors was a potential biomarker of thrombotic risk in patients with localized CRC (Zaragoza-Huesca et al., 2022). These observations have led our group to contemplate the contribution of hepsin to the hypercoagulable state of malignancy which, together with other clinical and biological risk factors that converge in cancer patients, ultimately promote the activation of the hemostatic system (Kim et al., 2020; Mahajan et al., 2022). Beyond our results about hepsin association with metastasis and thrombosis in CRC patients (Zaragoza-Huesca et al., 2022), we do not know the functional mechanisms underlying these links. In this study, we investigated the association of plasma hepsin levels with tumor stage and thrombosis in CRC patients. We examined the functional effects of hepsin expression on colon tumor cell proliferation, migration, invasion, activation of key cancer signaling proteins and plasma thrombin generation. As hepsin is a potential therapeutic target in CRC invasion and thrombogenesis, we performed a virtual screening (VS) to search for inhibitors that suppressed its protumor and prothrombotic effects *in vitro* and *in vivo*.

## 2 Materials and methods

### 2.1 Ethics statement

The study was conducted in accordance with Good Clinical Practice guidelines and the Declaration of Helsinki. This study was approved by the Ethics Committee of the Morales Meseguer University Hospital (EST: 07/15). All participants who were still alive during data collection provided written and signed informed consent.

The experiments complied with the Guidelines of the Council of the European Union (Directive 2010/63/EU) and RD 53/2013 of Spain. The experiments and procedures were performed as approved by the Counseling of Water, Agriculture, Livestock, and Fisheries of the Autonomous Community of the Region of Murcia (CARM authorization number # A13180602).

### 2.2 Samples and patient characteristics

Seventy-three patients with localized or advanced CRC were treated between 2012 and 2021 at Morales Meseguer Hospital University. Participants were recruited directly by an oncologist at their first appointment or during chemotherapy. All participants with advanced tumors had active disease at the onset. KRAS mutations were evaluated by real-time polymerase chain reaction (PCR) using the Idylla system (BioCartis).

### 2.3 Plasma hepsin determination by ELISA

Human hepsin ELISA (ELH-HPSN-1, RayBiotech) was performed to quantify human plasma hepsin (diluted 1:2) in patients with localized (*n* = 20) and metastatic (*n* = 53) CRC, according to the manufacturer’s instructions.

### 2.4 Cell culture

The cells were cultivated under standard conditions (37°C, 5% CO_2_ and >95% humidity). Caco-2 (RRID: CVCL_0025), DLD-1 (RRID: CVCL_0248), and HCT-116 (RRID: CVCL_0291) colorectal cancer cell lines were purchased from the American Type Culture Collection and authenticated as previously described (Peñas-Martínez et al., 2021). The cells were cultivated in MEM, RPMI and McCoy’s 5a medium containing 1% GlutaMAX, 20% fetal bovine serum (FBS) and penicillin/streptomycin (Gibco-Thermo Scientific). Cells were routinely tested for *mycoplasma* contamination.

### 2.5 Cell transfection

Caco-2 cells (10 × 10^6^ cells) were plated in 500 μL of the Ingenio electroporation solution (Mirus Vio). Cells were transfected with plasmid DNA pcMV6-AC (contains hepsin gene) or transfection control pCMV-MIR (Origene), and selected using antibiotic geneticin/G418 (Gibco-Thermo Scientific) for 10 days. After selection, single cells were plated into each well of a 96-well plate to obtain single clones.

### 2.6 RT-qPCR

Total RNA was obtained from Caco-2 (cells transfected with pCMV-MIR), Caco-2-HPN (hepsin-overexpressing cells transfected with pCMV6-AC), DLD-1, and HCT-116 cells using Trizol^®^ Reagent (Invitrogen). A NanoDrop spectrophotometer (Thermo Scientific) was used to determine the RNA concentration and the 260/280 ratio. From the total RNA, 100 ng/sample was reverse-transcripted to cDNA (SuperScript First Strand, Invitrogen). PCR was performed in triplicates using the TaqMan^®^ gene expression probes for hepsin (hs01056332_m1) and β-actin (hs01060665_g1) on a LC480 Real-Time PCR system (Roche). β-actin expression was used as an endogenous reference control using the comparative cycle threshold (Ct) method (2^−ΔΔCT^).

### 2.7 Western blot analysis

Cell lysates were subjected to 10% SDS PAGE and subsequently transferred to nitrocellulose membranes. Protein detection was performed using primary rabbit anti-human hepsin (Sigma Aldrich), phospho-Protein Kinase B (pAKT) (Invitrogen), phospho-extracellular signal-regulated kinases 1 and 2 (pERK1/2) (9101S, Cell Signaling Technologies, Werfen) and phospho-signal transducer and activator of transcription 3 (pSTAT3; Tyr705) (9145, Cell Signaling Technologies, Werfen) antibodies. Secondary IgG antibodies were horseradish peroxidase-coupled and visualized using the ECL kit. Protein expression of β-actin (Sigma-Aldrich) was used as an endogenous reference control.

### 2.8 Wound healing assays

Caco-2 and Caco-2-HPN cells were grown as confluent and wounded by removing a 300–500 μm-wide cell strip through the well with a standard 200 μL pipet tip. Wounded monolayers were washed twice to remove non-adherent cells. Wound healing was quantified after 72 h using ImageJ software as previously described (Peñas-Martínez et al., 2021).

### 2.9 Degradation of gelatin coated coverslips

The gelatin matrix was prepared by mixing 0.2% gelatin and rhodamine (Invitrogen, Life Technologies) as previously described (Luengo-Gil et al., 2016). Coverslips were coated with a gelatin mixture, fixed with 0.5% glutaraldehyde for 15 min, and washed with PBS. Caco-2 and Caco-2-HPN cells were cultured on coverslips for 72 h. Immunofluorescence analysis was performed after fixing the cells with 3.7% formaldehyde and incubating them with phalloidin (Sigma-Aldrich) and ProLong Gold Antifade medium with DAPI (ThermoFisher). Images were taken with a confocal spectral scanning microscope SP8 LEICA, analyzed with Fiji-ImageJ and GIMP software, and processed with Adobe Photoshop. Degraded gelatin cells were quantified as previously described (Luengo-Gil et al., 2016).

### 2.10 Proliferation assays

To evaluate the proliferative activity of Caco-2 and Caco-2-HPN cells, 5-ethynyl-2′-deoxyuridine (EdU) was added for 48 h and EdU-positive cells were detected by fluorescent-azide coupling reaction with EdU (Click-iT; Thermo Fisher Scientific) using a BD Accuri C6 flow cytometer.

### 2.11 Thrombin generation assay

Venous blood samples were collected from 20 healthy donors. Blood was drawn from the antecubital vein into non-siliconized Vacutainer tubes containing 3.8% buffered sodium citrate (Becton Dickinson). The tubes were centrifuged for 15 min at 2,000 g at room temperature, and platelet-poor plasma was prepared as previously described (Salta et al., 2018) and stored at −80°C.

Caco-2 and Caco-2-HPN cells were cultured as confluent monolayers in 6-well plates and incubated with 1 mL of plasma for 3 h at 37°C and 5% CO_2_. The plasma was recovered and centrifuged for 5 min at 200 g at room temperature. Thrombin generation assay-calibrated automated thrombogram (TAG-CAT) was performed as previously described (Pascreau et al., 2019) (Diagnostica Stago). The samples were processed in duplicate. The following thrombogram parameters were evaluated: a) lag time, b) time to peak (ttPeak), c) peak, d) mean rate index (MRI), and e) endogenous thrombin potential (ETP) (Pascreau et al., 2019).

### 2.12 Virtual screening

To identify novel and safe hepsin inhibitors, we performed a VS based on the molecular docking technique (Alburquerque-González et al., 2021) with Autodock Vina (Trott and Olson, 2010), processing the hepsin crystallographic structure with PDB code 1P57 against the DrugBank (https://go.drugbank.com) database of compounds [version 5.0; of 9,591 compounds, there are 2,037 approved by the American Food and Drug Administration (FDA), 96 nutraceuticals and 6,000 experimental], and focusing the docking process on its catalytic site with residues Histidine-57 (HIS57), Aspartic acid-102 (ASP102) and Serine-195 (SER195).

### 2.13 Effect of selected drugs on hepsin activity, cell migration, invasion, proliferation and thrombin generation

Recombinant hepsin (0.05 µM) (R&D Systems) was incubated in 50 mM Tris-HCl buffer (pH 9) with 200 µM BOC-Gln-Arg-Arg-AMC fluorogenic substrate, and the emitted fluorescence was recorded for 5 min. To test the effect of the selected drugs, the same reaction was performed, followed by incubation with hepsin for 1 h at 37°C. The IC50 was calculated after registering hepsin activity at different drug concentrations.

Cells were incubated in the presence or absence of 1.88 µM venetoclax to evaluate colorectal cancer cell migration, invasion, proliferation and thrombin generation under the same conditions described above.

### 2.14 Larval xenotransplantion assays

Wild-type zebrafish (*Danio rerio H. Cypriniformes*, *Cyprinidae*) were obtained from the Zebrafish International Resource Center (ZIRC). We used transparent *roya9/a9* and *nacrew2/w2* (casper) zebrafish, which have been previously described (Wenz et al., 2020). Fish were mated, staged, raised and processed, as described in the zebrafish handbook (Westerfield, 2000). Fertilized zebrafish eggs were obtained from the natural spawning of fish and were maintained in our facilities following standard husbandry practices. The animals were kept under a 12 h light/dark cycle at 28°C. Zebrafish larvae were anaesthetized as described previously (Wenz et al., 2020).

Caco-2-HPN cells were cultured in the presence or absence of 1.88 µM venetoclax for 48 h. Then, these cells and Caco-2 were disaggregated and labelled with 1,1′-di-octa-decyl-3,3,3′,3′-tetra-methyl-indo-carbo-cya-nine perchlorate (DiI, ThermoFisher) and finally resuspended in a buffer containing 5% FBS in PBS. Cell injection into embryos was conducted as previously described (Gabellini et al., 2018) and larvae were scored for cell dissemination by fluorescence microscopy. Caco-2 and Caco-2-HPN cell invasion scores were calculated as described previously (Gabellini et al., 2018).

### 2.15 Statistical analysis

The Aalen-Johansen estimator was used to evaluate thrombosis cumulative incidence. The analyses were performed using log-transformation of plasma hepsin concentration. The association between hepsin (continuous, log-transformed values) and thrombosis was examined using Fine-Gray regression. Furthermore, the third quartile (Q3) of hepsin concentration (log-transformed) was used to produce a stratified description of its association with thrombosis cumulative incidence. The hepsin distribution was compared using the Wilcoxon test. Time-to-thrombosis was defined as the time elapsed between the date of hepsin extraction and thrombosis, censoring event-free subjects and factoring in death as a competing event. Analyses were performed using R version 4.05 (R Core Team, 2014).

## 3 Results

### 3.1 Hepsin levels in plasma of CRC patients

**FIGURE 1:**
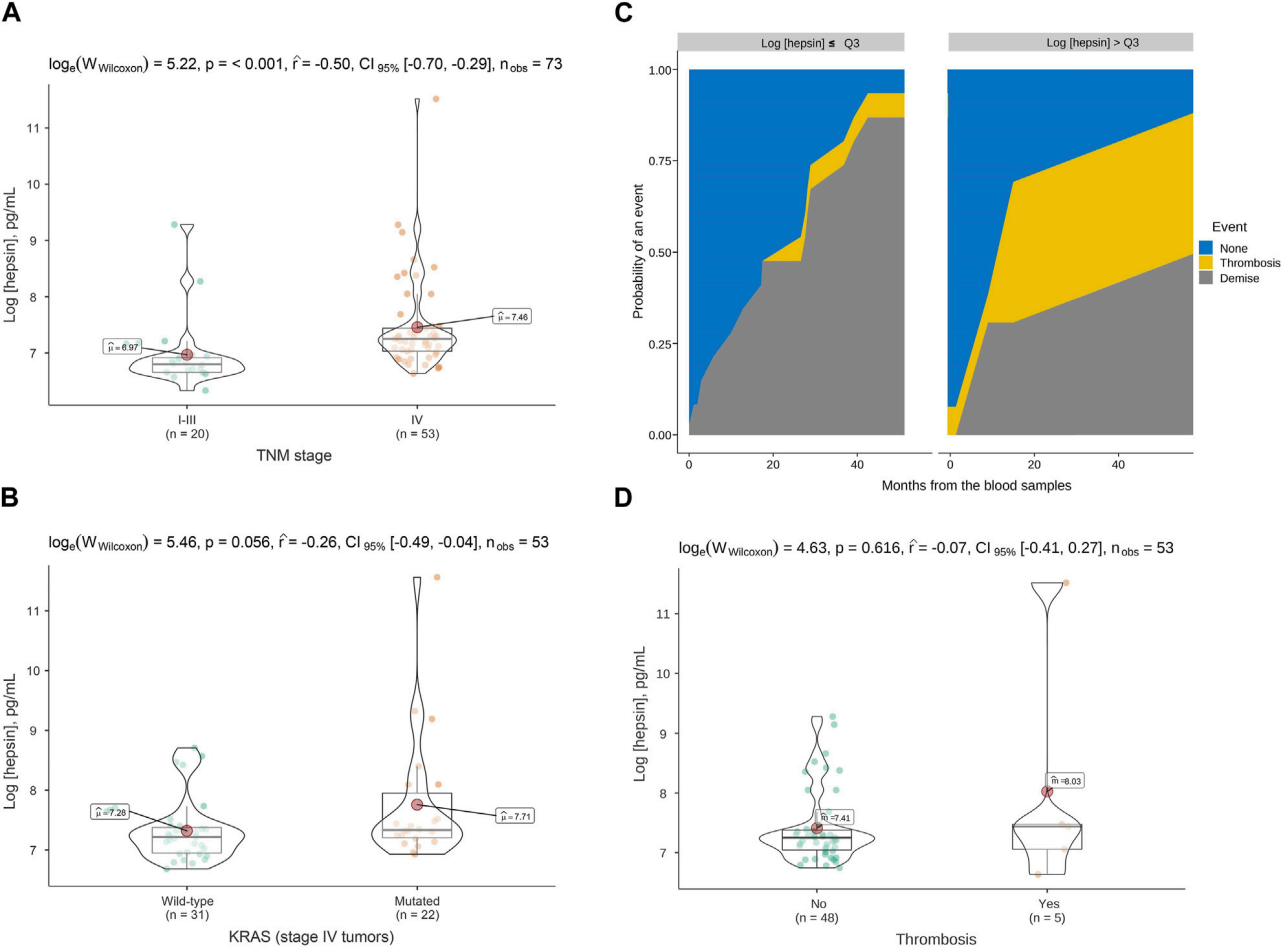
Hepsin levels in plasma of colorectal cancer patients and association with tumor stage, KRAS mutation and thrombosis. **(A)** Violin boxplot showing the association between the logarithm of the hepsin concentration and TNM stage, **(B)** and the presence of KRAS mutations in advanced cancer patients. **(C)** Cumulative incidence function for thrombosis among advanced cancer patients. The third quartile of the hepsin level distribution was used to stratify the curves. **(D)** Violin box-plots showing the log hepsin concentration in advanced cancer patients with or without thrombosis. *p*, *p*-value; *CI*, confidence interval; *n*_*obs*_, number of patients; *TNM*, tumor, node, metastases; *Q3*, third quartile.

We first evaluated plasma hepsin levels by ELISA in 73 CRC patients. Baseline patient characteristics are shown in Table 1. Of these patients, 73% had advanced tumors (*n* = 53) and 27% had localized neoplasms (*n* = 20). The mean natural logarithm of hepsin concentration was greater in advanced tumors than in localized tumors (7.4 vs. 6.9, *p*-value<0.001) (pg/mL) (Figure 1A). In advanced tumors (*n* = 53), the mean logarithm of hepsin concentration was 7.2 vs. 7.7 pg/mL in KRAS native and mutated tumors, respectively (*p*-value = 0.056) (Figure 1B). At the time of analysis of patients with advanced cancer, five thrombotic events were detected, comprising a cumulative incidence (CI) of 7.0% [95% confidence interval (CFI), 1.6–17.7].

**TABLE 1:** Baseline patient characteristics.

Characteristics	N (%)
Age. median (range)	63 (39–85)
Sex. male	49 (67.1)
TNM stage	
Localized	20 (27.3)
Advanced	53 (72.6)
Line of therapy	
1st line	22 (30.1)
2nd line	14 (19.1)
3rd line	10 (13.6)
4th line	5 (6.8)
Time from diagnosis of metastasis to hepsin sampling. median (range)	4.2 (0–34)
Location of the primary tumor*	
Right colon	28 (38.5)
Transverse colon	2 (2.6)
Left colon	31 (42.4)
Rectum	12 (16.4)
Location of metastases	
Liver	41 (56.1)
Lung	20 (27.3)
Lymph nodes	18 (24.6)
Bone	4 (5.4)
Peritoneal	19 (26.0)
Surgery of the primary tumor	58 (79.4)
Histological grade	
Grade 1	20 (28.7)
Grade 2	38 (52.0)
Grade 3	9 (12.3)
Unknown	5 (6.8)
KRAS	
Mutant	22 (30.1)
Wild-type	31 (42.4)
Not performed	20 (27.3)
Chemotherapy regimen when hepsin was obtained*	
FOLFOX-based	11 (15.0)
Irinotecan-based	9 (12.3)
Anti-angiogenic	13 (17.8)
Anti-EGFR	1 (1.3)
Regorafenib	2 (2.7)
None	44 (60.2)

*TNM*, tumor, node, metastases; *KRAS*, Kirsten rat sarcoma viral oncogene Homologue; *FOLFOX*, oxaliplatino-5-fluorouracil; *EGFR*, epidermal growth factor receptor; *: Not mutually exclusive.

Subjects with a logarithm of hepsin concentration >Q3 (7.4 pg/mL) had a 24-month thrombosis CI of 34.7% (95% CFI, 0.2-86) compared to zero events in subjects with levels ≤Q3 (*p*-value = 0.036, Gray test) (Figure 1C). However, the results were subjected to uncertainty due to the low number of events and the effect of an influential observation (Figure 1D). In the Fine-Gray regression, continuous hepsin (log-transformed) was associated with more thrombosis with a sub-hazard ratio of 2.12 (95% CFI, 1.50–2.98).

### 3.2 Hepsin levels affect Caco-2 cells invasion, but not migration and proliferation

**FIGURE 2:**
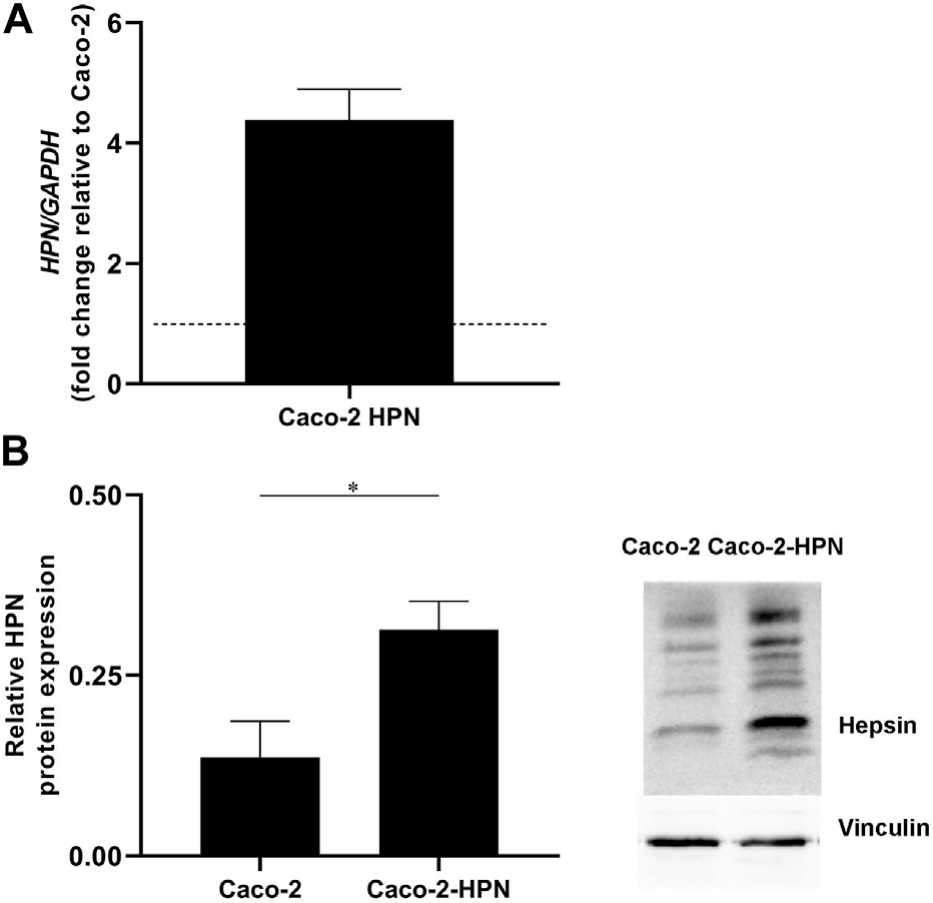
Hepsin overexpression in Caco-2-HPN cells. **(A)** The hepsin mRNA levels were deter-mined in Caco-2 and Caco-2-HPN cells by RT-qPCR. Gene expression levels were normalized to b-actin mRNA levels and the data are represented as the mean ± standard error of the mean of technical triplicates. Levels were shown as fold increase relative to the mean of Caco-2 cells. **(B)** Hepsin protein levels were determined in Caco-2 and Caco-2-HPN cells by electrophoresis and Western blot in lysates of Caco-2 and Caco-2-HPN cells. Vinculin expression was detected as loading control to relativize hepsin levels. Data are represented as the mean ± standard error of the mean of technical triplicates. *HPN*, Hepsin; *Caco-2-HPN*, Caco-2 cells overexpressing hepsin; *Caco-2*, Caco-2 cells with hepsin basal expression; *: *p*-value < 0.05.

Basal hepsin expression was low in CRC Caco-2 cells. Clones overexpressing hepsin were generated by stable transfection with the hepsin gene to boost expression. Caco-2-HPN exhibited a 4.4-fold increase in mRNA levels compared to Caco-2 (Figure 2A), which correlated with an effective increase in protein expression (Figure 2B).

**FIGURE 3:**
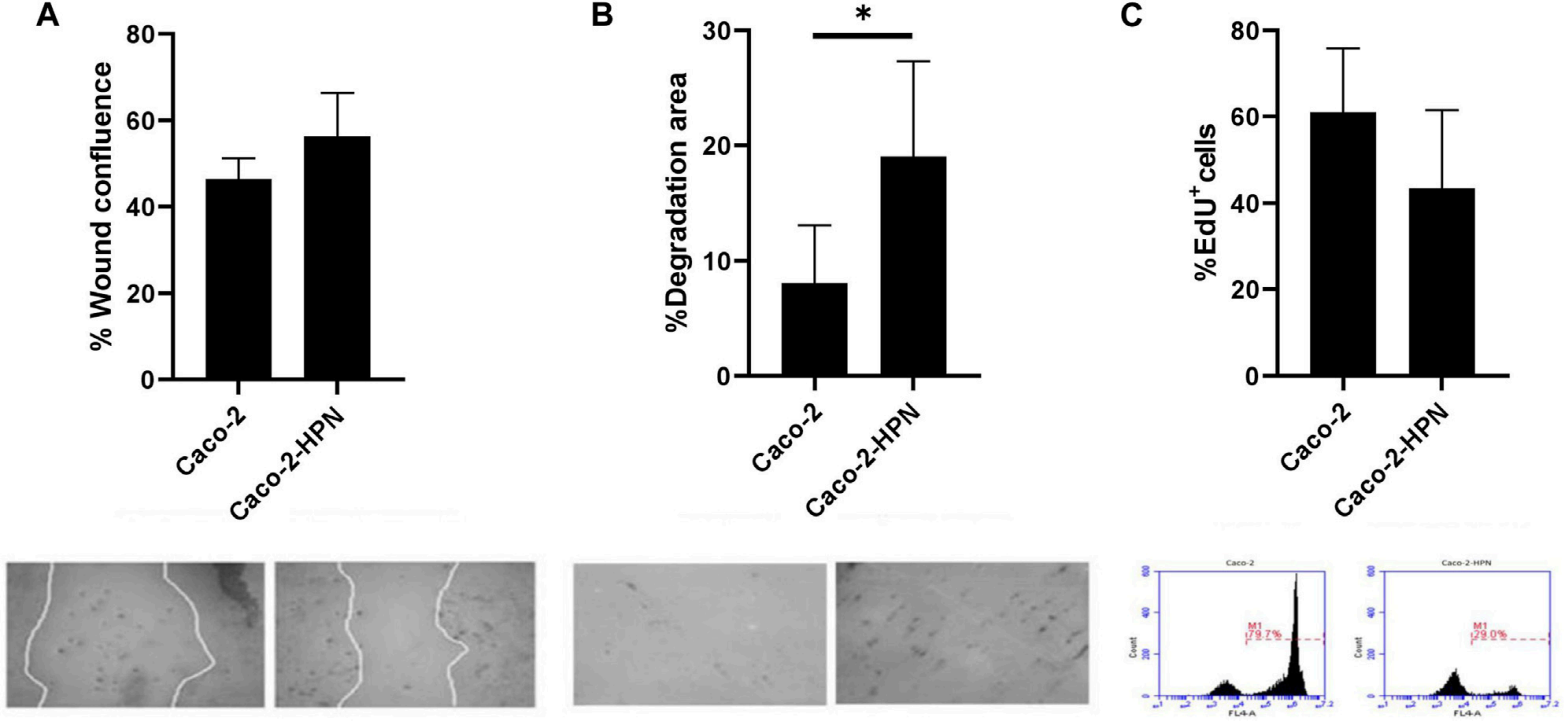
Effects of hepsin levels on cell migration, invasion and proliferation in Caco-2 and Caco-2-HPN cells. **(A)** Percentage of wound confluence was evaluated after 48 h of the wound created with a pipette tip in Caco-2 and Caco-2-HPN. Six different images were processed for each sample. Images were recorded with a Leica microscope at 5× and Fiji-ImageJ was used to analyze migration. The graph represents the mean ± standard error of the mean of the replicates. Under the graph, the white continuous lines represent the limits of the space without cell monolayers after 48 h of the wound. **(B)** Percentage of cells invading gelatin matrix was evaluated after 72 h of cell culture in the matrix. Images were taken with a confocal spectral scanning microscope SP8 LEICA, analyzed with ImageJ and GIMP software, and processed with Fiji-ImageJ. Six different images were processed for each sample. Cells that degraded gelatin were scored as positive. The graph represents the mean ± standard error of the mean of the replicates. Under the graph, black points on the bright matrix represent cells that have invaded or degraded gelatin. **(C)** The percentage of EdU positive cells was determined in Caco-2 and Caco-2-HPN cells by flow cytometry after 48 h of cell culture. Each condition was evaluated in triplicate. The graph represents the mean ± standard error of the mean of the replicates. Under the graph, we show representative plots from EdU assay by flow cytometry. Edu fluorescence was detected by FL4-A channel. *Caco-2-HPN*, Caco-2 cells overexpressing hepsin; *Caco-2*, Caco-2 cells with hepsin basal expression; *: *p*-value < 0.05; *EdU* ^*+*^, positive for 5-ethynyl-2′-deoxyuridine; *M1*, cells proliferating according to Edu.

Since enteropeptidase (another TTSP) expression regulates glioblastoma cell migration (Peñas-Martínez et al., 2021), we investigated the effect of hepsin on CRC cells. Our results are compatible with a slight increase in Caco-2-HPN cell migration versus Caco-2 cells (46.35% ± 4.87% vs. 56.31% ± 9.99%), although the difference lacks statistical significance (Figure 3A).

Since hepsin is a serine protease, we also analyzed cell invasion by examining the capacity of the cells to degrade a gelatin matrix. Interestingly, overexpression of hepsin significantly increased the ability of the cells to degrade gelatin compared to the basal expression (19.04% ± 8.28% vs. 8.03% ± 5.05%) (Figure 3B).

Additionally, we assessed the effect of hepsin on cell proliferation and found that hepsin overexpression did not increase cell proliferation (Figure 3C).

### 3.3 Pro-tumor hepsin signaling pathway

**FIGURE 4:**
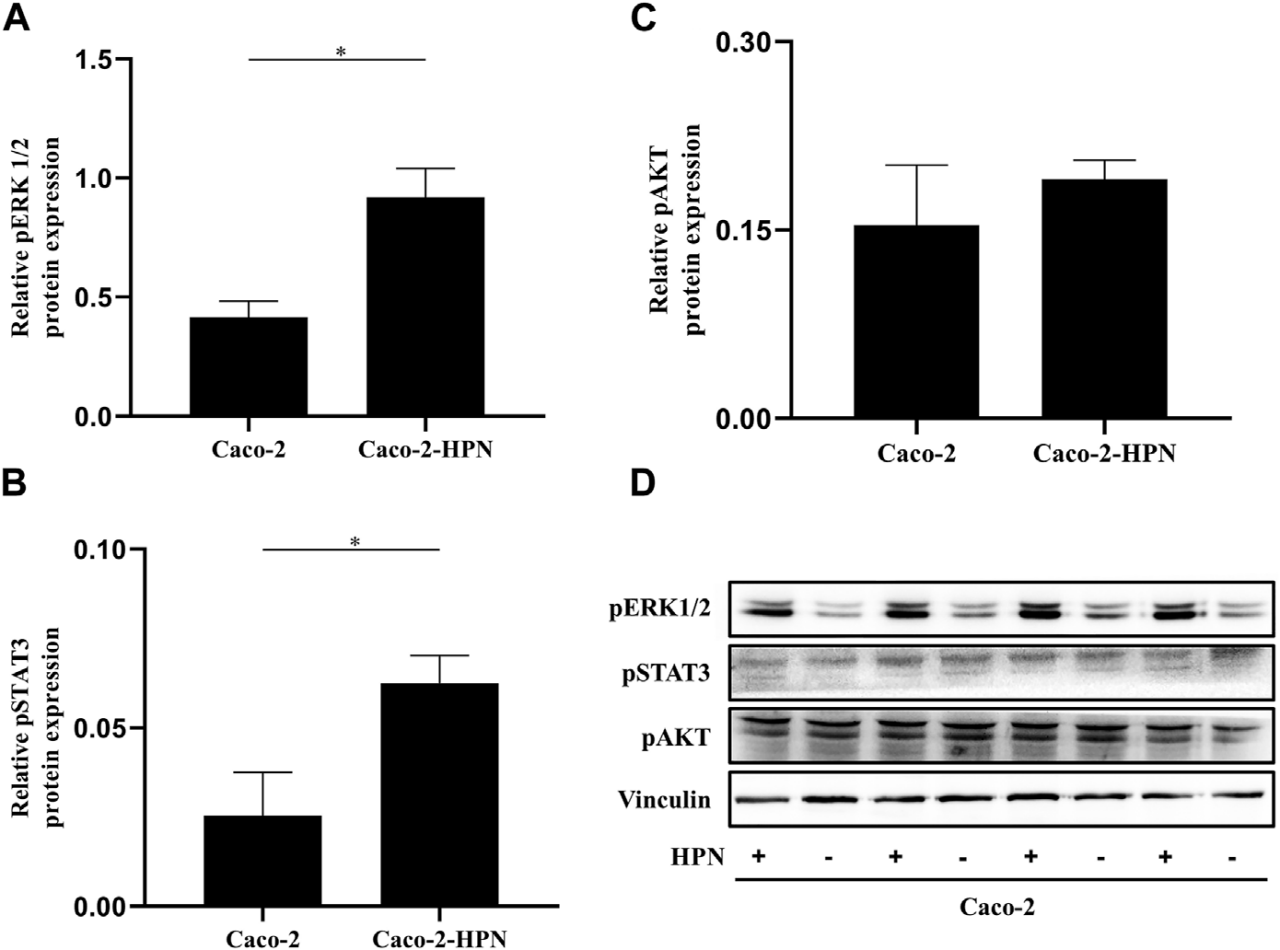
pSTAT3, pAKT and pERK1/2 expression in Caco-2 and Caco-2-HPN cells. Expression of pERK1/2 **(A)**, pSTAT3 **(B)** and pAKT **(C)** determined by electrophoresis and Western blot in lysates of Caco-2 and Caco-2-HPN cells in triplicates. Levels were determined by densitometry and represented as relative protein expression to vinculin. Graphs represent the mean ± standard error of the mean of the triplicates. **(D)** Electrophoresis and Western blot of pSTAT3, pAKT and pERK1/2 in lysates of Caco-2 and Caco-2-HPN cells in triplicates. Vinculin expression was detected as loading control. *pERK1/2*, phospho-extracellular signal-regulated kinases 1 and 2; *pSTAT3*, phospho-signal transducer and activator of transcription 3; *pAKT*, phospho-Protein Kinase B; *Caco-2-HPN*, Caco-2 cells overexpressing hepsin; *Caco-2*, Caco-2 cells with hepsin basal expression; *: *p*-value < 0.05; *HPN*, Hepsin; *+*, overexpression; *-*, basal expression.

Amplification of signaling mediated by Erk1/2 has favoured hepatic metastases of CRC. Therefore, we explored the effects of hepsin expression on this pathway. Figures 4A, D shows that in Caco-2 cells, hepsin overexpression was associated with higher Erk1/2 phosphorylation (2.2 times greater than basal expression). Likewise, hepsin overexpression resulted in greater phosphorylation of STAT3 (2.2 times greater than basal expression) (Figures 4B, D). However, cells overexpressing hepsin did not increase Akt phosphorylation (Figures 4C, D).

### 3.4 Thrombin generation assay

**FIGURE 5:**
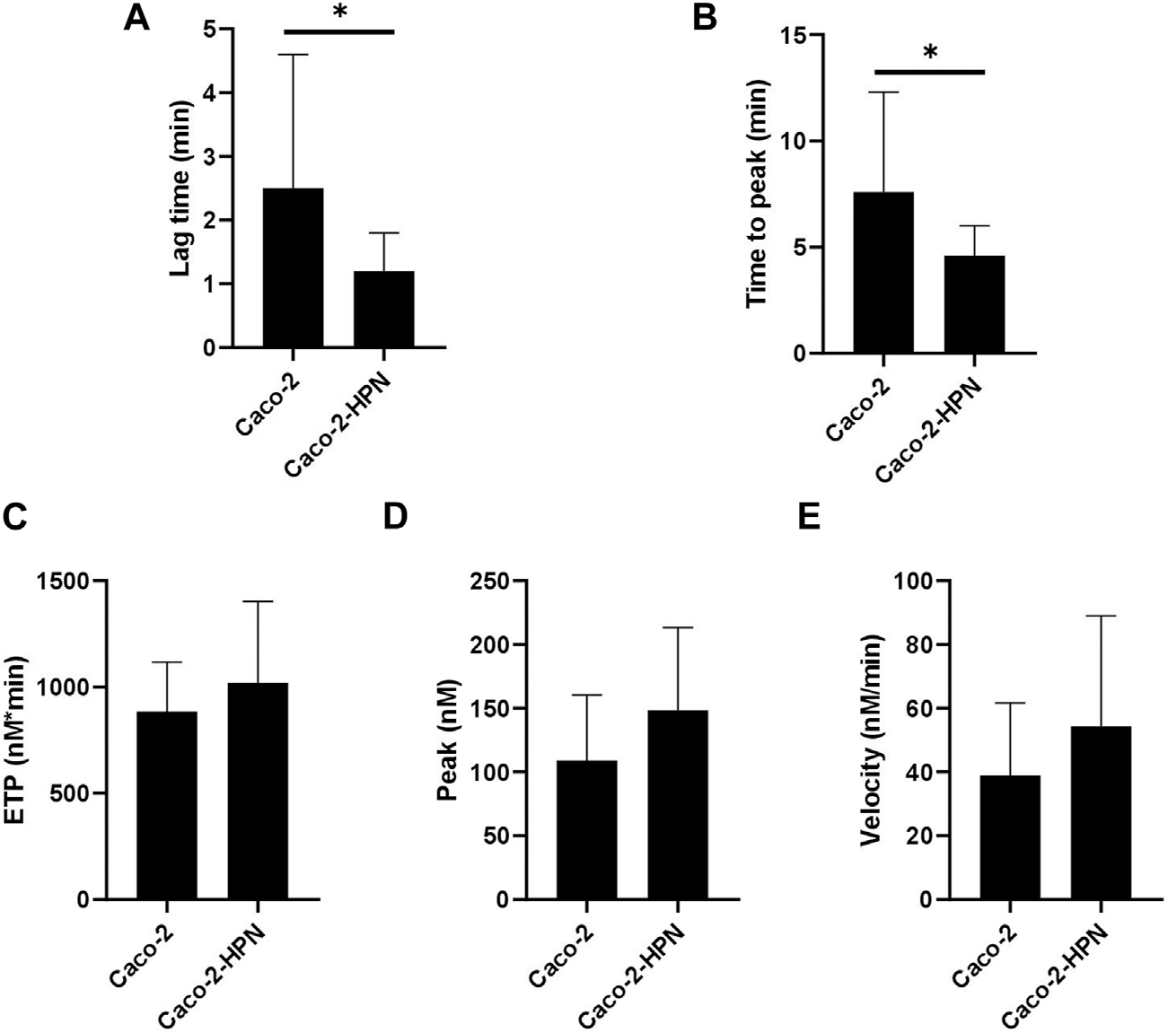
Effects of hepsin levels on thrombin generation by Caco-2 and Caco-2-HPN cells. Thrombin generation was performed after incubation of plasma with cells for 3 h as described in Materials and Methods. Afterwards, plasma was incubated with PPP reagent ^®^ (final concentrations: tissue factor, 1 pmol/L; phospholipids, 4 μmol/L) and calcium chloride. The lag time (min) **(A)**, time to peak (min) **(B)**, endogenous thrombin potential (ETP, nmol*min) **(C)**, thrombin peak (peak, nmol) **(D)**, and mean rate index (Velocity, nmol/min) **(E)** were recorded. The data represent the mean ± standard error of the mean of at least six separate experiments. *Caco-2-HPN*, Caco-2 cells overexpressing hepsin; *Caco-2*, Caco-2 cells with hepsin basal expression; *: *p*-value < 0.05.

We then investigated whether high hepsin levels could contribute to the generation of a procoagulant state, and hence, greater thrombotic risk. Therefore, we performed thrombin generation tests using plasma samples from healthy subjects incubated with Caco-2-HPN and Caco-2 cells. Exposure to Caco-2-HPN was associated with shorter lag time (Figure 5A) and ttPeak (Figure 5B) in comparison with Caco-2 cells, being these differences statistically significant (Table 2), whereas ETP (Figure 5C), thrombin peak (Figure 5D) and MRI (Figure 5E) showed no significant differences between the two types of cells (Table 2).

**TABLE 2:** Thrombin generation parameters in plasma preincubated with basal and hepsin overexpressing Caco-2 cells.

Thrombin generation parameters	Mean ± SEM Caco-2	Mean ± SEM Caco-2 HPN	*p*-value
ETP (nM*min)	884.5 ± 232.3	1,021 ± 382.6	0.22
Peak (nM)	109.1 ± 51.4	148.5 ± 65.0	0.06
Lag time (min)	2.5 ± 2.1	1.2 ± 0.6	0.043
ttPeak (min)	7.6 ± 4.7	4.6 ± 1.4	0.022
Mean rate index (nM/min)	38.9 ± 22.8	54.4 ± 34.6	0.16

*ETP*, endogenous thrombin potential; *ttPeak*, time-to-peak; *SEM*, standard error of mean; *Caco-2-HPN*, Caco-2 cells overexpressing hepsin; *Caco-2*, Caco-2 cells with hepsin basal expression.

### 3.5 Virtual screening

**FIGURE 6:**
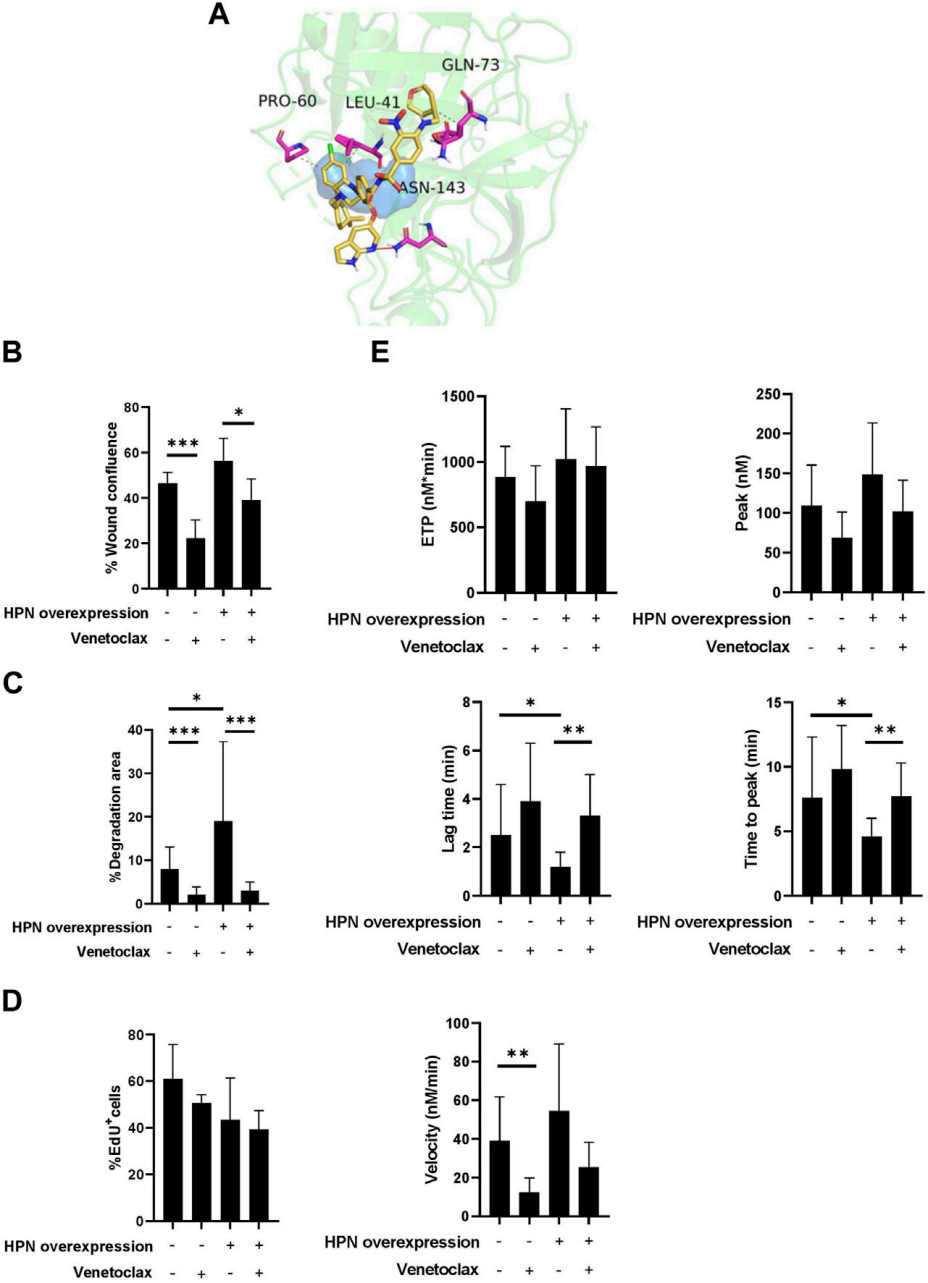
Venetoclax reduces protumor and prothrombotic effects of hepsin in colorectal cancer cells. **(A)** Obtained pose from molecular docking between venetoclax and hepsin. Venetoclax is shown in yellow skeleton, while hydrophobic interactions are represented in dashes, and hydrogen bonds with a red line. Catalytic triad is shown in blue surface, and hepsin amminoacids interacting with venetoclax are represented in fuchsia. **(B)** Percentage of wound confluence was evaluated after 48 h of the wound created with a pipette tip in Caco-2 and Caco-2-HPN, in the presence and absence of 1.88 µM venetoclax. Six different images were processed for each sample. Images were recorded with a Leica microscope at 5× and Fiji-ImageJ was used to analyze migration. The graph represents the mean ± standard error of the mean of the replicates. **(C)** Percentage of cells invading gelatin matrix were evaluated after 72 h of cell culture in the matrix, in presence and absence of 1.88 µM venetoclax. Images were taken with a confocal spectral scanning microscope SP8 LEICA, analyzed with ImageJ and GIMP software, and processed with Fiji-ImageJ. Cells that degraded gelatin were scored as positive. The graph represents the mean ± standard error of the mean of the replicates. **(D)** The percentage of EdU positive cells was determined in Caco-2 and Caco-2-HPN cells by flow cytometry after 48 h of cell culture in presence and absence of 1.88 µM venetoclax. The graph represents the mean ± standard error of the mean of the replicates. **(E)** Thrombin generation was performed after incubation of plasma with cells in presence and absence of 1.88 µM venetoclax as described in Materials and Methods. The endogenous thrombin potential (ETP, nmol*min), thrombin peak (peak, nmol), lag time (min), time-to-peak (min) and mean rate index (velocity, nmol/min) were recorded. The data represent the mean ± standard error of the mean of at least six separate experiments. Each condition was evaluated in triplicate. *PRO60*, proline-60; *LEU41*, leucine-41; GLN73, glutamine-73; *ASN143*, asparagine-143; *HPN*, Hepsin; *EdU* ^*+*^, positive for 5-ethynyl-2′-deoxyuridine; *: *p*-value < 0.05; **: *p*-value < 0.01; ***: *p*-value < 0.001.

Once the VS calculations were completed, the compounds were ranked according to their docking scores, and the top eight compounds were retained for posterior visual analysis. Venetoclax (DrugBank code DB11581) was selected and prioritized with a docking score of −11 kcal/mol. Additional selection criteria were hydrophobic interactions with residues proline-60 (PRO60), leucine-41 (LEU41) and glutamine-73 (GLN73) and their hydrogen bonds with asparagine-143 (ASN143) (Figure 6A), where venetoclax impedes access to the catalytic triad, as shown in the surface representation of its residues.

### 3.6 Venetoclax inhibition of hepsin

We tested the effect of venetoclax on hepsin by evaluating its proteolytic activity toward fluorogenic substrates. Venetoclax irreversibly inhibited hepsin activity. Therefore, we calculated the half-maximal inhibitory concentration (IC50) by incubating the protein with different concentrations of venetoclax and monitoring hepsin activity. As shown in Supplementary Figure S1, the IC50 of venetoclax was 0.48 μM, which reflected a better hepsin inhibition than the one recently reported for indole derivatives (Blay et al., 2020). The toxicity of these compounds has yet to be tested in humans, while the calculated 2xIC50 of venetoclax for hepsin is within the concentration range tested in chronic lymphocytic leukemia cells (Anderson et al., 2016).

### 3.7 Venetoclax effect on CRC cells

Venetoclax significantly reduced cell migration both in cells with basal expression (46.35% ± 4·87% vs. 22.26% ± 7·97%) as well as in cells that overexpressed hepsin (56.31% ± 9.99% vs. 39.09% ± 9.27%) (Figure 6B; Supplementary Figure S2). Venetoclax was also able to specifically inhibit Caco-2 and Caco-2-HPN cell invasion to a statistically significant degree (2.08% ± 1.81% vs. 8.03% ± 5.05% and 2.98% ± 2.03% vs. 19.04% ± 18.28%, respectively) (Figure 6C; Supplementary Figure S2). Although venetoclax is a Bcl-2 inhibitor, it did not affect CRC cell proliferation (Figure 6D). Finally, incubating the cells with venetoclax reduced the prothrombotic phenotype of both Caco-2 and Caco-2-HPN cells. We highlight a statistically significant reduction of the rate of thrombin generation in Caco-2 cells and a prolonged lag time and time to peak in Caco-2-HPN cells (Figure 6E; Table 3; Supplementary Figure S3). These antithrombotic effects were not observed in other CRC cells without hepsin expression, such as DLD-1 and HCT-116 cells (Supplementary Figures S4, S5; Supplementary Table S1).

**TABLE 3:** Thrombin generation parameters in plasma preincubated with basal and hepsin overexpressing Caco-2 cells in the presence and absence of venetoclax.

	Mean ± SEM				*p*-value	
	Caco-2	Caco-2 + Venetoclax	Caco-2- HPN	Caco-2-HPN + Venetoclax	Caco-2 vs. Caco-2 + Venetoclax	Caco-2-HPN vs. Caco-2-HPN + Venetoclax
ETP (nM*min)	884.5 ± 232.3	697.3 ± 271.9	1,021 ± 382.6	967.1 ± 299	0.14	0.78
Peak (nM)	109.1 ± 51.4	68.71 ± 32.5	148.5 ± 65	101.7 ± 39.7	0.11	0.15
Lag time (min)	2.5 ± 2.1	3.9 ± 2.4	1.2 ± 0.6	3.3 ± 1.7	0.21	0.001
ttPeak (min)	7.6 ± 4.7	9.8 ± 3.4	4.6 ± 1.4	7.7 ± 2.6	0.35	0.003
Mean rate index (nM/min)	38.9 ± 22.8	12.4 ± 7.4	54.4 ± 34.6	25.2 ± 13	0.022	0.09

*SEM*, standard error of mean; *Caco-2-HPN*, Caco-2 cells overexpressing hepsin; *Caco-2*, Caco-2 cells with hepsin basal expression; *ETP*, endogenous thrombin potential; *ttPeak*, time to peak.

### 3.8 Venetoclax effect on CRC cells invasiveness *in vivo*

**FIGURE 7:**
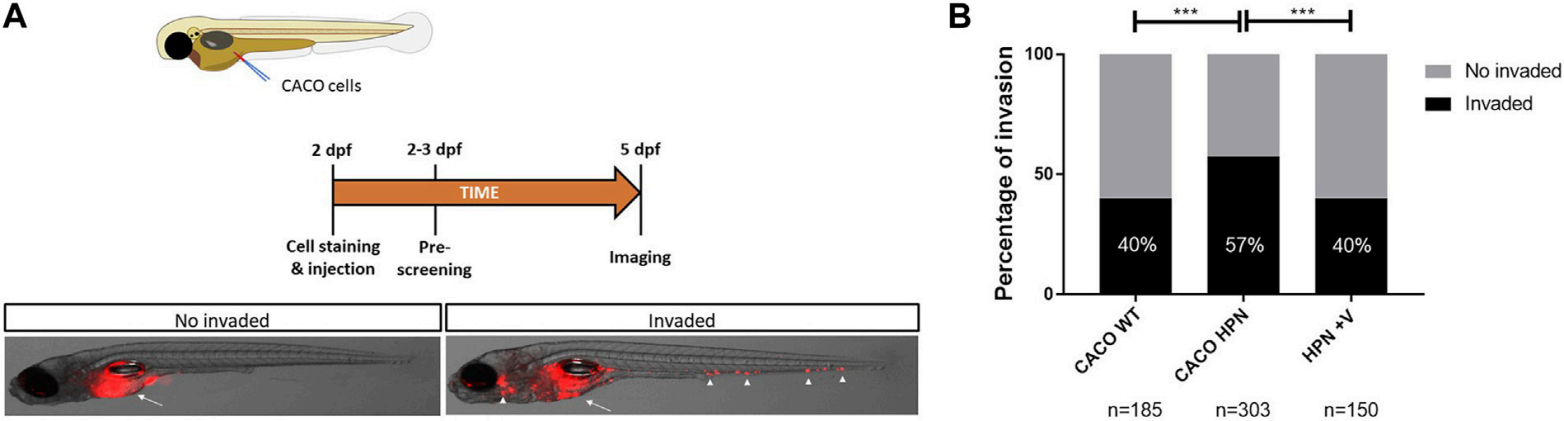
Venetoclax reduces Caco-2-HPN cell invasion in zebrafish larvae. **(A)** Experimental design of the zebrafish larvae xenotransplantation experiments and representative pictures of no invaded and invaded larvae showing red-labelled Caco-2 cells at the injection site (arrow) and several invasion foci (arrowheads). **(B)** Evaluation of invasion score 3 days after injection in the yolk sac of 2-dpf casper zebrafish larvae. The results showed are a pool of three different experiments. *dpf*, days post-fertilization; ***: *p*-value < 0.001; *n*, number of larvae per treatment; *CACO WT*, Caco-2 cells with hepsin basal expression; *CACO HPN*, Caco-2 cells overexpressing hepsin; *HPN + V*, Caco-2 cells overexpressing hepsin pretreated with venetoclax.

The higher *in vitro* invasiveness of Caco-2-HPN was confirmed *in vivo* by using a xenotransplantation model in zebrafish larvae. Interestingly, pretreatment of these cells with venetoclax reduced their invasiveness to levels found in Caco-2 cells (Figure 7), confirming the results of the *in vitro* studies and further showing its therapeutic potential for the treatment of CRC.

## 4 Discussion

Hepsin is a TTSP that exerts a complex influence on different types of cancer (Zhang et al., 2016; Damalanka et al., 2021; Tervonen et al., 2021; Lu et al., 2022), parallel to the activation of the hemostatic system, and comprises critical nodes where multiple signaling pathways converge with coagulation cascade effectors. CRC is a suitable model to explore these interactions owing to the downstream regulation of hepsin, which depends on the Ras/Erk1/2 signaling pathway (Tervonen et al., 2021), as well as the worse prognosis and greater thrombotic risk associated with the activation of this signaling pathway (Ades et al., 2015; Ottaiano et al., 2020). Recently, we identified hepsin from primary tumor as a prognostic marker for metastasis and thrombosis in patients with localized tumors (Zaragoza-Huesca et al., 2022). Interestingly, although the serine protease domain of hepsin is located in the extracellular region, some articles support its secretion, making it possible to monitor hepsin levels in the blood (Wang et al., 2019). This enabled us to evaluate it as a possible prognostic or predictive biomarker of tumor stage and thrombotic risk in the plasma. In addition, the role of hepsin in CRC remains to be elucidated. This led us to explore the possible hepsin-mediated mechanisms that might be linked to CRC tumorigenesis and thrombotic risk and to search for drugs that target this TTSP.

Our results in plasma from patients suggest that hepsin overexpression is associated with a metastatic phenotype, which corroborates the findings of Tieng et al. (2020), who reported elevated serum hepsin levels in patients with metastatic CRC, prompting its use as a soluble biomarker. Hepsin has an extracellular fraction that can be released from the transmembrane region of tumor cells (Wang et al., 2019). The fact that hepsin is differentially expressed in the plasma of metastatic patients could be due to its release by cells from metastatic niches and not from the primary tumor, where the role of hepsin would no longer be relevant (Willbold et al., 2019). Although the statistical evidence is limited, our results are consistent with earlier observations that implicate hepsin in processes related to Ras-dependent tumorigenesis (Tervonen et al., 2021). Therefore, the association between hepsin levels and Ras-dependent tumorigenesis should be reviewed considering the soluble hepsin fraction.

The proteolytic enzymes involved in metastasis might also account for the two-way links between thrombosis and cancer (Carmona-Bayonas et al., 2019; Kim et al., 2020; Mahajan et al., 2022), in which the degradation of the extracellular matrix by proteases is coupled to activating the hemostatic system (Fernandes et al., 2019). In our plasma series from patients, the incidence of thromboembolic disease was low, consistent with the literature (Riedl et al., 2017; Rees et al., 2018). Despite anecdotal evidence, it is striking that none of the metastatic individuals with hepsin levels ≤Q3 exhibited a thrombotic event in the first 24 months, whereas in metastatic patients with hepsin levels >Q3, 24-month thrombosis CI was 34.7%. These results could be explained by our *in vitro* experiments, where hepsin increased thrombin generation promoted by CRC cells, probably because of its capacity to activate coagulation factor VII (Halabian et al., 2009; Khandekar and Jagadeeswaran, 2014). Interestingly, in our published study, increased hepsin expression from primary tumor was a potential biomarker of thrombosis in patients with localized CRC, but not in metastatic ones (Zaragoza-Huesca et al., 2022). The fact that hepsin is not associated with an increased thrombotic risk in biopsies from metastatic patients could also be due to its delivering in plasma, since it is no longer useful in a tumor which has already invaded distant tissues. The results obtained in the plasma of the patients and *in vitro* in thrombin generation assays, together with the fact that thromboembolic diseases are often associated with metastasis (Carmona-Bayonas et al., 2019), led us to hypothesize that some TTSPs, including hepsin, contribute to hypercoagulability *in vivo* by generating thrombin at the boundary phase of the tumor invasion and migration front, together with other mediators (Razak et al., 2018; Reddel et al., 2019). To shed light on the mechanisms underlying tumorigenesis, we studied the influence of hepsin in CRC cell lines. Our results confirmed that hepsin expression was associated with increased CRC cell invasion. This is in line with findings in other pathologies, such as prostate cancer, in which hepsin proteolyzes laminin-332, a cell matrix molecule necessary for cell-to-cell adhesion, thereby enhancing tumor cell invasion (Pant et al., 2018). These actions are exerted directly or by inducing and activating matrix metalloproteinases, resulting in potent extracellular matrix destruction (Wilkinson et al., 2017). Hepsin is not the only TTSP involved in CRC invasion and metastasis. The upregulation of other key TTSPs, such as tissue‐type plasminogen activator, urokinase-type plasminogen activator, plasminogen activator inhibitor type‐1, and others, play an essential role in CRC tumorigenesis (Heissig et al., 2021; Sakamoto et al., 2021; Zhai et al., 2022). Together, they comprise a complex, redundant and multistage system that determines the degradation of the basement membrane and extracellular matrix by proteases.

Moreover, we found that hepsin overexpression was associated with greater Erk1/2 phosphorylation and STAT3 activation. Erk1/2-mediated signaling amplification has been shown to promote hepatic metastases in CRC (Urosevic et al., 2020). Interestingly, proteins that reduce Erk1/2 activation, such as CMTM4, have shown anti-tumor effects in CRC and other types of cancer (Xue et al., 2019). Simultaneously, the JAK2/STAT3 signaling pathway plays an important role in regulating apoptosis and enhancing clonogenic potential (Park et al., 2019). The mechanism underlying hepsin’s activation of these pathways remains unknown and this is a matter that merits study in the future. Hepsin can cleave the epidermal growth factor receptor (EGFR) so that the fragments are tyrosine-phosphorylated. However, it is not clear which downstream signaling pathways are activated since hepsin-induced EGFR cleavage does not correlate with increased Erk1/2 activation (Chen and Chai, 2017).

Due to pro-invasive and prothrombotic effects of hepsin, we also investigated its role as a potential therapeutic target in CRC. Initially, we silenced hepsin expression using siRNAs (Supplementary Figure S6); however, as the migration and invasion experiments took 72 h, transient silencing was gradually reversed and no statistical differences were observed between Caco-2 and Caco-2-HPN cells. Therefore, a different strategy was implemented. To this end, we used VS to identify the potential hepsin inhibitors. We identified venetoclax, a drug used in combination to treat acute myeloid leukemia and relapsed or refractory chronic lymphocytic leukemia (DiNardo et al., 2018; Seymour et al., 2018). We demonstrated that venetoclax inhibited the protumoral and prothrombotic effects of hepsin in CRC cells. It is important to note that venetoclax also exerts these inhibitory effects on Caco-2 cells because these cells also exhibit basal hepsin expression. Venetoclax had no clear effect on thrombin generation in CRC cells lacking hepsin expression. Surprisingly, although venetoclax is a BCL2 inhibitor, an anti-apoptotic protein that is pathologically overexpressed and crucial to the survival of certain cancer cells, Caco-2 cell proliferation was not affected by treatment with this drug. This may be due to drug efflux pumps preventing it from reaching the inside of the cell (Giacomini et al., 2010), although this was not investigated in the present study. Overall, these observations support the notion that the antitumor effect of venetoclax in Caco-2 cells occurs in the extracellular space via hepsin inhibition. Most importantly, in zebrafish larvae, venetoclax significantly reduced the invasion of hepsin-overexpressing cells to the levels found in parental cells, demonstrating its efficacy in an *in vivo* model. Although other hepsin inhibitors have been reported, their usefulness is limited by their decreased stability *in vivo*. Moreover, some drugs exhibit off-target actions that diminish their inhibitory effectiveness (Damalanka et al., 2018).

Our study had limitations that should be borne in mind. The scant samples did not allow for a solid analysis of the association between plasma hepsin and thrombotic risk in metastatic patients. As with any other biomarker, interpreting the effect of its levels is complex because it is contingent on specific molecular pathways (e.g., the mitogen-activated protein kinase pathway) and presumably depends on circumstances such as tumor burden, tumor response to chemotherapy, or antagonistic or paradoxical effects (Halabian et al., 2009; Kim et al., 2020; Mahajan et al., 2022). Consequently, the results should be interpreted as hypothesis generators, which are reviewed later. First, there is no correlation between the findings found in the plasma and biopsies of patients with CRC (Zaragoza-Huesca et al., 2022), and more studies are needed to determine the potential of hepsin as a biomarker and therapeutic target in patients with metastasis. Second, although this study sheds light on the status of two key pathways in CRC (Erk1/2 and JAK2/STAT3 signaling pathways), the causal order is unclear. Third, our research revealed the antitumor and potentially anti-thrombotic effects of venetoclax, an FDA-approved oral agent, possibly due to its anti-hepsin activity. However, it cannot be ruled out that this effect might be mediated by its action on other proteins. Identifying patients with CRC who could benefit from venetoclax in clinical trials is also necessary.

In conclusion, our results demonstrated that high hepsin levels are associated to a more aggressive tumor phenotype because of the higher potential for invasion of CRC cells and greater activation of coagulation. Furthermore, we identified hepsin as a novel therapeutic target for these malignancies and their underlying pathologies. In this context, this study reports, for the first time, an anti-migratory, anti-invasive and anti-thrombotic effect of venetoclax, which could indicate its use as a new molecular-targeted treatment for CRC and other hepsin-overexpressing tumors.

## Data Availability

The raw data supporting the conclusion of this article will be made available by the authors, without undue reservation.
