# Supplementary material for: Venetoclax is a potent hepsin inhibitor that reduces the metastatic and prothrombotic phenotypes of hepsin-expressing colorectal cancer cells
Source: Front Mol Biosci. 2023 May 19;10:1182925. doi: 10.3389/fmolb.2023.1182925 (PMC10235687; doi:10.3389/fmolb.2023.1182925)
Supplement: Supplementary file 1 [file DataSheet1.docx]

Supplementary Material

**Venetoclax is a potent hepsin inhibitor that reduces the metastatic and prothrombotic phenotypes of hepsin-expressing colorectal cancer cells.**

Maria Carmen Rodenas, Julia Peñas-Martínez, Irene Pardo-Sánchez, David Zaragoza-Huesca, Carmen Ortega-Sabater, Jorge Peña-García, Salvador Espín, Guillermo Ricote, Sofía Montenegro, Francisco Ayala-de la Peña, Ginés Luengo-Gil, Andrés Nieto, Francisco García-Molina, Vicente Vicente, Francesco Bernardi, María Luisa Lozano, Victoriano Mulero, Horacio Pérez-Sánchez*, Alberto Carmona-Bayonas*, Irene Martínez-Martínez*

*** Correspondence:** Horacio Pérez-Sánchez: [hperez@ucam.edu](mailto:hperez@ucam.edu); Alberto Carmona-Bayonas: [alberto.carmonabayonas@gmail.com](mailto:alberto.carmonabayonas@gmail.com); Irene Martínez-Martínez: [immlgi@um.es](mailto:immlgi@um.es).

# Supplementary Figures and Tables

## Supplementary Figures


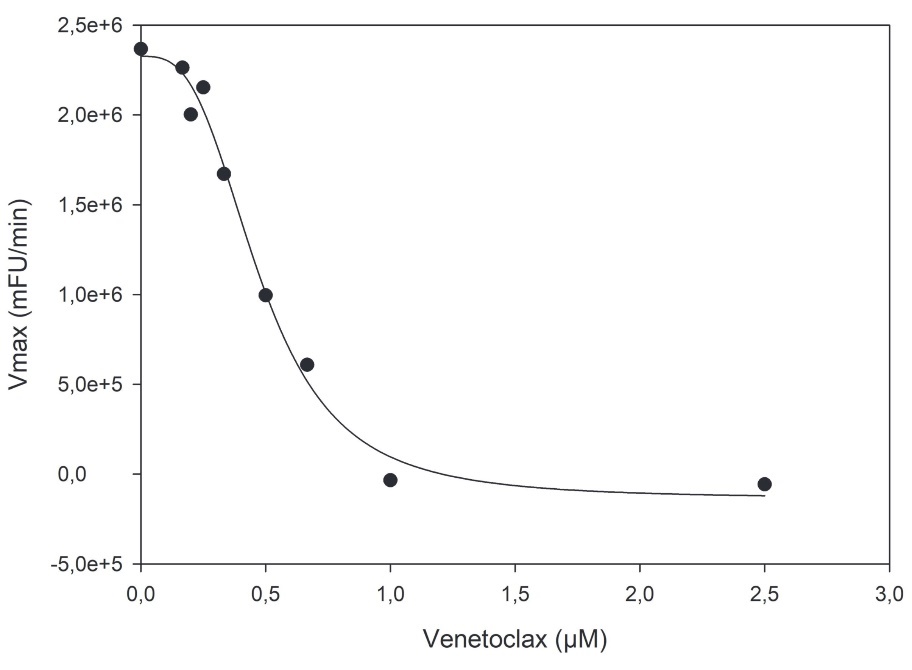


**Supplementary Figure 1. Irreversible inhibition of hepsin proteolytic activity by venetoclax.** *Vmax*: Maximum velocity; *FU*: Fluorescence units.

**Supplementary Figure 2. Effects of venetoclax on Caco-2 and Caco-2-HPN migration and invasion.** Upper images were recorded with a Leica microscope at 5× and Fiji-ImageJ was used to analyze migration. In the upper images, the white continuous lines represent the limits of the space without cell monolayers after 48 hours of the wound. Inferior images were taken with a confocal spectral scanning microscope SP8 LEICA, analyzed with ImageJ and GIMP software, and processed with Fiji-ImageJ. In the inferior images, black points on the bright matrix represent cells that have invaded or degraded gelatin. *Caco-2-HPN*: Caco-2 cells overexpressing hepsin; *Caco-2*: Caco-2 cells with hepsin basal expression; *VEN*: Venetoclax.


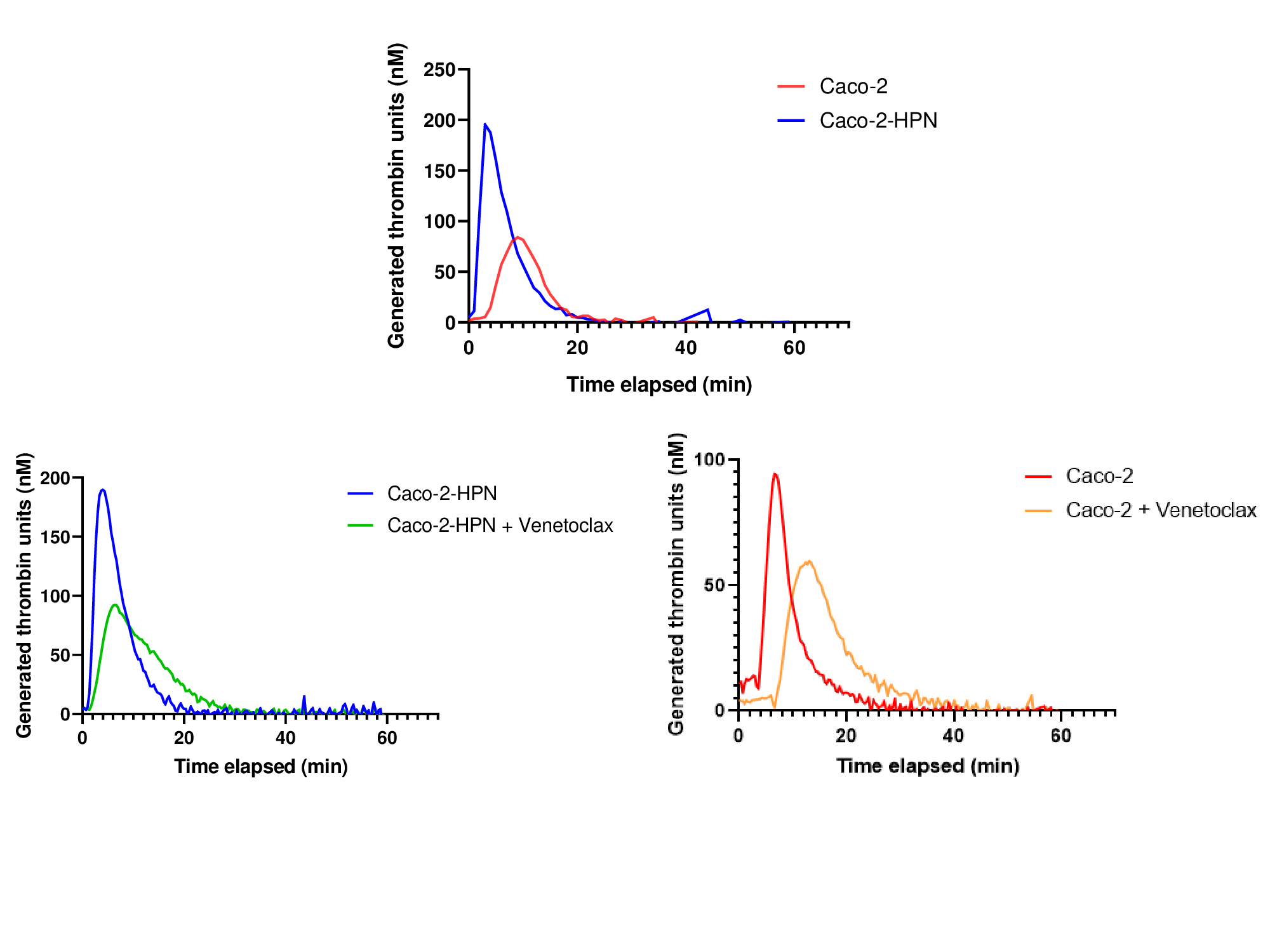


**Supplementary Figure 3. Representative thrombograms of plasma preincubated with Caco-2 and Caco-2-HPN cells in absence and presence of venetoclax.** *Caco-2-HPN*: Caco-2 cells overexpressing hepsin; *Caco-2*: Caco-2 cells with hepsin basal expression.

**
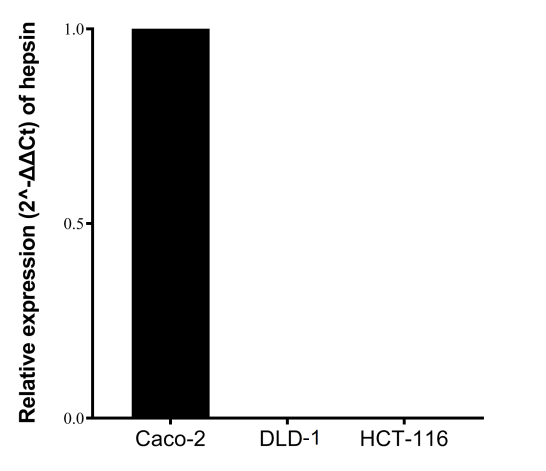
**

**Supplementary Figure 4. Relative expression (2^[-ΔΔCt]) of hepsin RNAm in DLD-1 and HCT-116 cell lines compared to Caco-2 expression.** β-actin expression was used as an endogenous reference control.


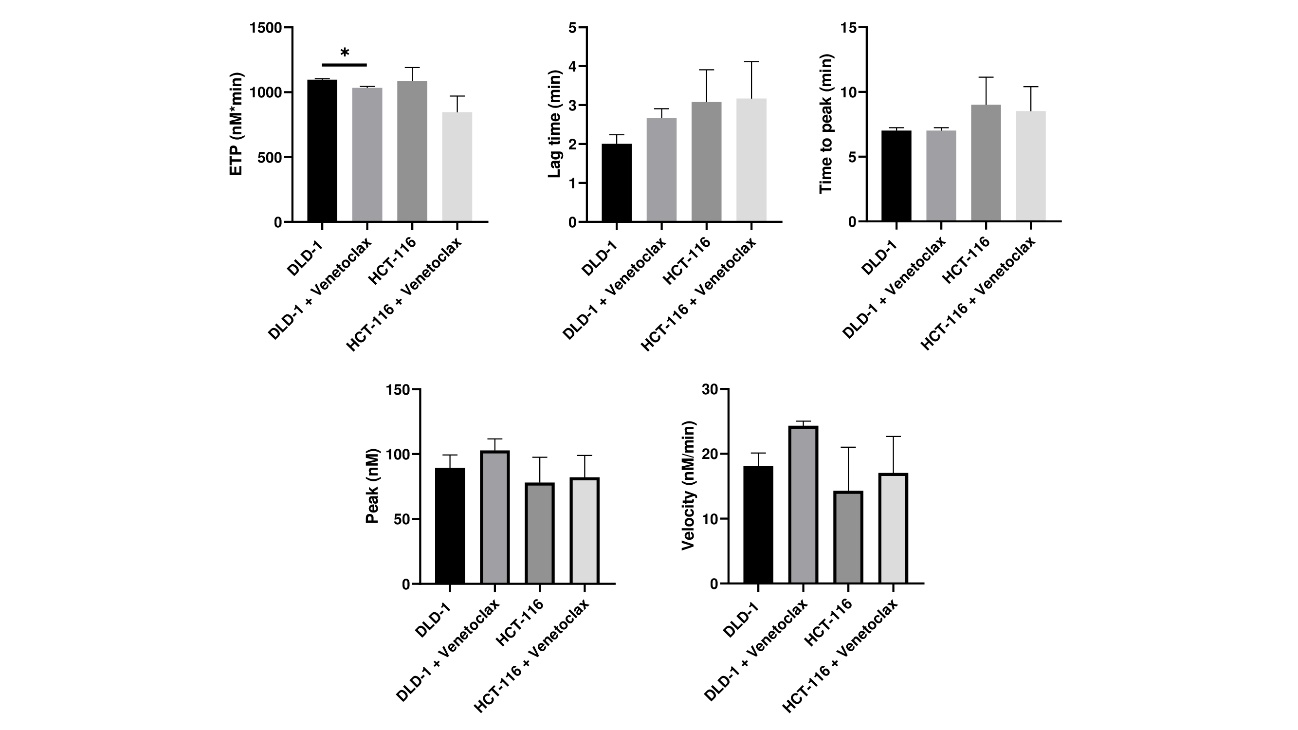


**Supplementary Figure 5. Effects of Venetoclax on thrombin generation promoted by DLD-1 and HCT-116 cells.** Thrombin generation was performed after incubation of plasma with cells for 3h as described in Materials and methods. Afterwards, plasma was incubated with PPP reagent ® (final concentrations: tissue factor, 1 pmol/L; phospholipids, 4 μmol/L) and calcium chloride. The endogenous thrombin potential (ETP, nM*min), thrombin peak (peak, nM), lag time (min), time to peak (min) and mean rate index (Velocity, nM/min) were recorded. The data represent the mean ± standard error of the mean of at least four separate experiments. ***: p-value<0.05.


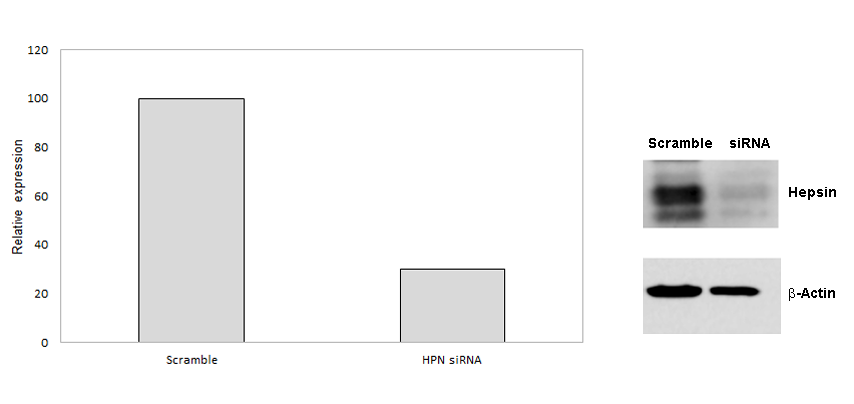


**Supplementary Figure 6. Hepsin gene silencing efficiency in Caco-2 cells after 24 hours of transfection**. ON-TARGETplus SMARTpool siRNAs against hepsin and control siRNAs were obtained from Dharmacon (GE Dharmacon, Barcelona, Spain). siRNA transfections on Caco-2 cells were performed using PepMute transfection reagent (SignaGen, MD, USA). On the left, we measure hepsin RNAm in cells transfected wit hepsin siRNAs, relativizing it to cells transfected with control siRNAs. On the right, hepsin protein levels were determined by electrophoresis and western blot in lysates of Caco-2 transfected with control or 5 nM siRNAs against HPN. Beta-actin expression was used as a loading control for both RNAm and protein detection. *Relative expression*: percentage of hepsin RNAm considering 100% in cells with siRNAs control; *Scramble*: cells transfected with siRNAs control; *HPN siRNA*: cells transfected with siRNAs against hepsin.

## Supplementary Tables

**Supplementary Table 1. Thrombin generation parameters in plasma preincubated with DLD-1 and HCT-116 cells in the presence and absence of Venetoclax.** *SEM*: standard error of mean; *ETP*: endogenous thrombin potential.

|  | **Mean ±SEM** | | | | **p-value** | |
| --- | --- | --- | --- | --- | --- | --- |
|  | DLD-1 | DLD-1 + Venetoclax | HCT-116 | HCT-116 + Venetoclax | DLD-1 vs DLD-1 + Venetoclax | HCT-116 vs HCT-116 + Venetoclax |
| **ETP (nM*min)** | 1095.695±8.309 | 1032.665±12.325 | 1087.405±101.901 | 845.39±124.917 | 0.0267 | 0.1677 |
| **Peak (nM)** | 89.330±10.041 | 102.835±8.846 | 78.020±19.516 | 82.075±16.907 | 0.2897 | 0.8449 |
| **Lag time (min)** | 2.005±0.233 | 2.67±0.240 | 3.085±0.827 | 3.17±0.948 | 0.1069 | 0.9326 |
| **Time to peak (min)** | 7.015±0.233 | 7.015±0.233 | 9.015±2.128 | 8.515±1.888 | >0.9999 | 0.8269 |
| **Mean rate index (nM/min)** | 18.115±2.001 | 24.310±0.721 | 14.295±6.725 | 17.03±5.643 | 0.0542 | 0.7025 |
